# Cleanroom‐Free Toolkit for Patterning Submicron‐Resolution Bioelectronics on Flexibles

**DOI:** 10.1002/smll.202411979

**Published:** 2025-03-07

**Authors:** Xudong Tao, Alejandro Carnicer‐Lombarte, Antonio Dominguez‐Alfaro, Luke Gatecliff, Ji Zhang, Sophia Bidinger, Scott T. Keene, Salim El Hadwe, Chaoqun Dong, Alexander J. Boys, Christopher Slaughter, Ruben Ruiz‐Mateos Serrano, Jakob Chovas, Marco Vinicio Alban‐Paccha, Damiano Barone, Sohini Kar‐Narayan, George G. Malliaras

**Affiliations:** ^1^ Electrical Engineering Division Department of Engineering University of Cambridge Cambridge CB3 0FA UK; ^2^ Department of Materials Science and Metallurgy University of Cambridge Cambridge CB3 0FS UK; ^3^ Bainbridge Bio Ltd The Hauser Forum 3 Charles Babbage Rd Cambridge CB3 0GT UK; ^4^ Department of Materials Science and NanoEngineering Rice University Houston TX 77005 USA; ^5^ Thayer School of Engineering Dartmouth College Hanover NH 03755 USA; ^6^ Department of Chemical Engineering and Biotechnology University of Cambridge Cambridge CB3 0AS UK; ^7^ Department of Medicine Addenbrooke's Hospital University of Cambridge Cambridge CB2 2QQ UK; ^8^ Department of Clinical Neurosciences Cambridge Biomedical Campus Cambridge CB2 0QQ UK; ^9^ Department of Neurosurgery Houston Methodist Houston 77030 USA

**Keywords:** flexible bioelectronics, submicron patterning, two‐photon laser

## Abstract

Fabricating flexible bioelectronics remains an ongoing challenge in pursuing a cost‐effective, efficient, scalable, and environmentally friendly approach for research and commercial applications. The current dominant method, lithography, presents challenges due to its incompatibility with solvent‐sensitive biomaterials and the phase mismatch between the photoresist and flexible substrates, such as elastomers. This study proposes a simplified, cleanroom‐free toolkit as a potential alternative to lithography for fabricating intricate bioelectronics on flexible substrates with submicron resolution. This technique integrates a two‐photon laser writing mask, mask transfer, and multi‐layer/material patterning processes, enabling batch‐to‐batch processing and making it suitable for scalable production. With excellent conformal patterning capability, different functional and encapsulation biomaterials can be patterned on flexible substrates, including elastomers, parylene‐C, polymer sheets, skin, fabric, and plant leaves. The versatility of this toolkit is validated by fabricating various prototypes of wearable and implantable bioelectronics, demonstrating excellent performance.

## Graphical abstract

This study presents a cleanroom‐free toolkit for patterning submicron‐resolution flexible bioelectronics, combining two‐photon laser writing, mask transfer, and multi‐layer patterning assembly. This technique enables precise and scalable patterning of diverse biomaterials on flexible substrates, offering the potential for developing wearable and implantable bioelectronics with excellent functionality and performance.

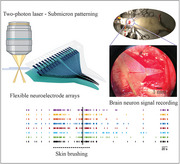

## 1 Introduction

Bioelectronics, the integration of biology and electronics,^[^ ^1^ ^]^ is revolutionizing bioengineering and healthcare by advancing the development of wearable and implantable devices as well as in vitro biological applications.^[^ ^2^ ^]^ Bioelectronics integrates concepts from electronics, materials science, and engineering to develop innovative technologies for interfacing with biological systems. Fabricating bioelectronic devices requires careful consideration of essential factors, including material selection, patterning resolution, device intricacy, biocompatibility, immune response, long‐term stability, and device performance.^[^ ^3^, ^4^ ^]^

The lithography technique remains the predominant method in bioelectronics fabrication,^[^ ^5^, ^6^, ^7^ ^]^ enabling the creation of micro‐ and nanoscale electronics on flexible substrates. However, challenges persist in terms of cost‐efficiency, environmental impact, and compatibility with both biomaterials and flexible substrates. The complexity and high cost of lithography^[^ ^8^ ^]^ limit its scalability, reducing the efficiency of academic research and impeding bioelectronics commercialization. The large quantities of photoresist and solvent required in lithography raise significant environmental concerns,^[^ ^9^ ^]^ and they are incompatible with sensitive biomaterials, such as proteins, which degrade under solvents and UV exposure.^[^ ^10^, ^11^ ^]^ The elastomeric substrate, e.g., polydimethylsiloxane (PDMS) is crucial in flexible bioelectronics due to its comparable stiffness to that of tissue,^[^ ^12^, ^13^ ^]^ and ease of fabrication to thicknesses necessary for brain tissue penetration–essential for high‐quality recordings in neural devices.^[^ ^14^, ^15^, ^16^ ^]^ However, lithography requires an exceptionally flat substrate surface for mask transfer,^[^ ^17^ ^]^ limiting its application on rough surfaces such as PDMS, where the phase mismatch between the photoresist and PDMS can lead to temperature‐induced cracking.^[^ ^18^ ^]^ Although lithography achieves nanometer‐scale resolutions, most bioelectronic applications do not require such precision; sub‐micron resolution is often adequate and can even prevent impedance mismatch, which affects device functionality.^[^ ^19^, ^20^ ^]^ Besides lithography, direct laser‐assisted carbonization^[^ ^21^ ^]^ is emerging as a promising fabrication technique for micro‐resolution bioelectronics,^[^ ^22^, ^23^, ^24^ ^]^ particularly by simplifying production processes and reducing costs.^[^ ^25^ ^]^ This approach involves applying a laser beam to induce thermal decomposition of precursor materials, such as polymers or organic compounds, forming carbon‐based structures.^[^ ^26^ ^]^ However, it is limited to carbon‐based materials. Therefore, there is a strong case for focusing on submicron‐scale bioelectronics fabrication,^[^ ^8^ ^]^ where sufficiently precise resolutions can be achieved, simplifying the process, improving cost‐effectiveness, minimizing environmental impact, and enabling compatibility with a broader array of biomaterials and substrates.

In this study, we propose a simplified, cleanroom‐free, direct laser writing‐assisted toolkit for fabricating intricate bioelectronics with submicron resolution on flexibles. This technique integrates direct laser writing mask, mask transfer, and multi‐layer/material patterning processes. It replaces the traditional lithography process by using a µm‐resolution patterned PaC mask instead of photoresist, light exposure, and solvents, thereby minimizing environmental impact and expanding applicability to more biomaterials. This toolkit enables the fabrication of submicron‐scale devices with intricate geometry and multiple materials (metal, functional, and encapsulation) on flexible substrates such as PDMS, PaC, thin polymer sheets, skin, fabric, and plant leaf, all in a cleanroom‐free manner. It also demonstrates excellent conformal patterning capability, enabling patterning across both edged and curved surfaces. We demonstrate the versatility of this toolkit by fabricating and validating a range of bioelectronics prototypes, including implantable neuroelectrode arrays, wrist pulse wearables, aptamer‐based biosensors, and organic electrochemical transistors (OECTs).

## 2 Toolkit for Fabricating Bioelectronics

**Figure 1:**
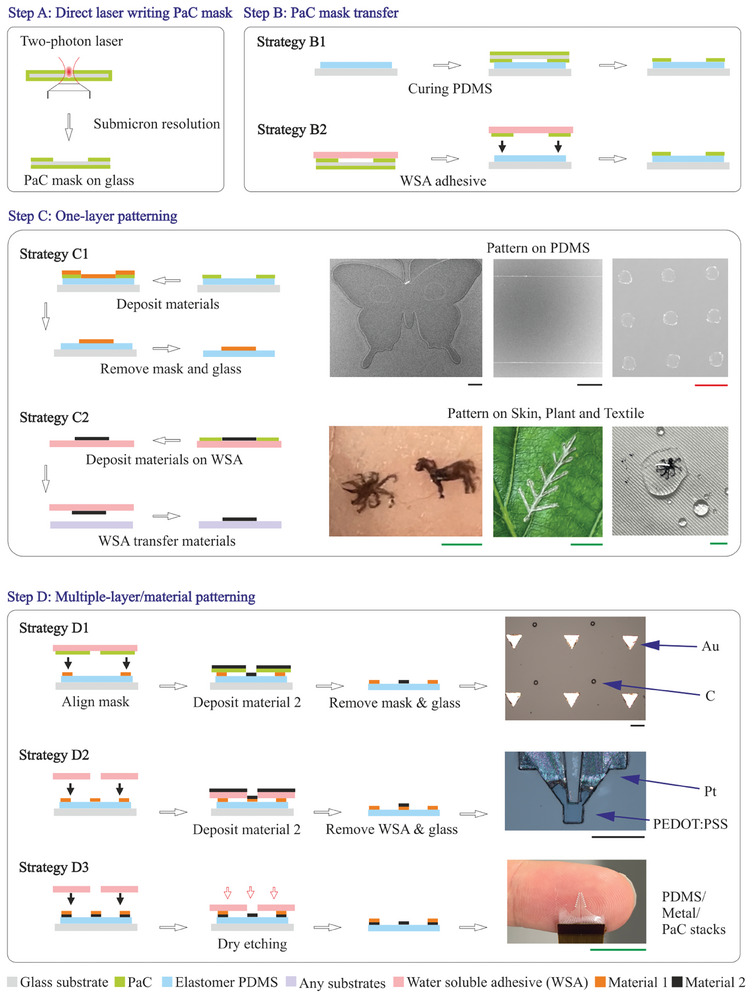
Toolkit for integrating bioelectronics (see Figure S2 for examples, Supporting Information). The red scale bar is 3 µm, the black scale bar is 100 µm and the green scale bar is 5 mm.

The essential toolkit is shown in **Figure** **1**. The fabrication processing includes direct laser writing mask (Step A), mask transfer (Step B), materials deposition (Step C), and assembly of intricate geometry with multiple materials and layers (Step D). The fabrication processes are conducted outside of a cleanroom, with air blowing applied to the sample surface before each step to remove potential dust. We proposed several strategies for each step, tailored to the specific application requirements such as size, materials, resolution, geometry, and application of the device, with selection guidance provided in Figure S1 (Supporting Information). These processes establish a versatile, cleanroom‐free toolset for fabricating bioelectronics on flexible substrates, accommodating diverse complex geometries with resolutions down to submicron scale. Next, we will discuss these steps in detail (see Figure 1; Figures S1 and S2, Supporting Information for each step).

1. Step A: The process begins with the direct laser writing mask using either a two‐photon laser on PaC for submicron resolution, or a CO_2_‐based laser on polyimide sheets (Kapton) for millimeter resolution. The two‐photon laser source can achieve nanometre‐scale resolution, while the ablation on PaC limits the pattern resolution to the submicron level. PaC is an ideal mask material due to its easily controlled thickness during chemical vapor deposition (CVD), good thermal resistance for laser ablation, and its relatively high modulus and hydrophobic properties which aid in the mask transfer process. It is crucial to optimize the PaC thickness between 1 and 2 µm during CVD deposition; if too thick, laser ablation through the material becomes challenging, while too thin prevents successful substrate detachment. To ensure ease of the peel‐off of the PaC mask from the glass substrate, the glass is soaped prior to PaC deposition.
2. Step B: To transfer the PaC mask onto flexible substrates, two methods were proposed: the PDMS curing process (Strategy B1) and the water‐soluble adhesives (Strategy B2). With Strategy B2, a water‐soluble adhesive (WSA, PVA‐based 3M 5414) peels the PaC mask from the glass substrate and transfers it to the flexible substrate. After dissolving the WSA, the PaC mask stays on the flexible substrate, though the initial mask‐substrate contact may be suboptimal. To improve the mask‐substrate contact, Strategy B1 involves the PDMS curing process. PDMS is spin‐coated with controlled thickness onto a PaC‐coated glass substrate for easier peel‐off. Before curing the PDMS, the PaC mask on glass is placed atop the uncured PDMS. The vacuum process removes air gaps between the PDMS and PaC mask, followed by thermal curing. Once PDMS is cured, the glass substrate for the PaC mask is removed, leaving the PaC mask adhered to the PDMS. This method ensures a strong mask‐substrate contact, allowing for adjustable PDMS thickness tailored to various bioelectronic applications. Importantly, the PaC mask can be peeled off from the PDMS substrate afterward. This approach, where PDMS is cured on the PaC mask, contrasts with the method of depositing PaC via CVD onto cured PDMS, where the deposited PaC is difficult to remove from the PDMS. Strategies B1 and B2 differ in terms of mask‐substrate contact, hence the choice between them depends on whether the material deposition process is dry or wet in the next step.
3. Step C: Following mask transfer, the next step is materials deposition. For cases where mask contact is less critical, dry deposition processes (such as physical vapor deposition, PVD) are suitable. Alternatively, for processes requiring good mask contact, both dry and wet processes can be used, e.g., PVD and spinning coating. Subsequently, material patterns are achieved by peeling off the PaC mask (Strategy C1). In contrast, Strategy C2 allows for the direct deposition of materials onto the mask/WSA. After peeling off the mask, the patterned materials on WSA can be transferred to substrates such as skin, PDMS, plant leaf, and fabric. After dissolving the WSA, the deposited materials remain on the substrate, necessitating that the materials themselves have sufficient adhesion to adhere effectively. It is important to note that this method (Strategy C2) necessitates a water‐free environment during the materials deposition to prevent premature dissolution of the WSA.
4. Step D: Devices with multiple materials and layers can be patterned using a PaC mask combined with WSA for additive materials processing (i.e., Strategy D1), or using a WSA mask alone for additive/subtractive processing (i.e., Strategy D2 or D3). All these strategies utilize WSA, offering the advantages of being contact gap‐free and residual‐free, compared to flexible masks and tape masks. This step involves using a micron‐level aligner to precisely align the PaC/WSA mask with the previously deposited patterns. As shown in Figure S2 (Supporting Information), the Finetech aligner enables micron‐resolution alignment with precise adjustments in the x‐y directions, while simultaneously imaging the alignment process using a top‐view camera with z‐axis focus adjustment for the two parts. As demonstrated in Step D (Figure 1), the WSA‐assisted mask can be attached to the glass slide, simplifying the alignment process and ensuring the mask remains flat. The sample on PDMS remains on the hard glass substrate throughout the fabrication process until all fabrication steps are completed. Following alignment, the WSA‐assisted approach ensures that the mask stays on the PDMS surface. Once the fabrication process is complete, the PDMS substrate can be removed from the glass slide, facilitated by the previously deposited parylene layer on the glass slide. The PaC mask achieves submicron resolution using a two‐photon laser, while WSA can be shaped with a CO_2_‐based laser cutter at millimeter resolution. Due to the mask‐substrate contact and the water‐soluble nature of WSA, dry deposition processes like PVD are recommended for Strategies D1 and D2. For Strategy D3, subtractive processing requires materials that can be etched using reactive ion etching. The backside of the WSA mask can be coated with hard materials such as metal or stuck onto polyimide tape to enhance its resistance during reactive ion etching. In addition to metal and functional materials, encapsulation patterning layers, e.g., PaC or PDMS can also be patterned using this strategy.

## 3 Materials Properties

The toolkit offers multiple strategies for each step, with the selection criteria detailed in Figure S1 (Supporting Information). All the strategies have been validated, as demonstrated in Figure S3 (Supporting Information), or through the examples of bioelectronics presented below. These validations highlight the effectiveness of the methods in achieving the desired outcomes, with each strategy successfully contributing to the fabrication of functional devices. The patterning capabilities of the toolkit were validated across different substrates, including elastomer, polymer film, skin, fabric, and plant leaf, using various fabrication techniques as summarized in Figure S1 (Supporting Information). Using PDMS elastomer as a substrate allows for adjustable thickness by varying the spin‐coating rate, catering to diverse wearable and implantable applications. In Figure S3a,e (Supporting Information), implant‐grade materials such as Au, Pt, and PEDOT: PSS can be patterned on PDMS using Step B1/2 + C1. Wearable‐grade conductive materials like eutectogels can be blade‐printed onto mask/WSA using Step C2 and transferred onto skin and textiles as tattoos. This is a substrate‐free approach that creates conductive tattoos on WSA, which can then be transferred to any substrate followed by dissolving the WSA. Ag nanoparticles and Pt (PVD) can be patterned directly using a WSA mask (Step D2) on plant leaves and polymer sheets, allowing for pattern sizes beyond wafer dimensions, and offering significant advantages over traditional lithography techniques. Due to the ultra‐flexibility of the PaC mask, the WSA‐assisted process with Steps D1 enables conformal patterning across both edged and curved surfaces. The materials demonstrate excellent flexibility during bending and stretching while maintaining electrical functionality (Figure S3b, Supporting Information). The eutectogels tattoos on the skin resist air blowing and can be removed with flowing water, and the fabric patterns also resist air blowing (Figure S4, Supporting Information), while the leaf patterns exhibit strong adhesion even after drying (Figure S3a, Supporting Information).

The patterning resolution is influenced by the laser source and the mask materials/thickness. A two‐photon laser can achieve a resolution as fine as 100 nm.^[^ ^27^ ^]^ Theoretically, the patterning resolution can match the two‐photon laser's capability of 100 nm. However, this is limited by the thickness of the PaC due to the aspect ratio effect during laser ablation on PaC. While a thinner PaC layer improves resolution, it must be thick enough to be peeled off; empirically, a minimum thickness of 1 µm is necessary. In our process, the PaC thickness was controlled to 2 µm to facilitate mask transfer onto PDMS. High‐density arrays were fabricated using Step B1 + C1 by optimizing the laser slicing number, i.e., the number of laser ablations on PaC (see Figures S5 and S6, Supporting Information). For a 2 µm thick PaC mask layer, a slicing number of 17 achieved electrode arrays with a 1 µm width and 29 µm spacing, maintaining high transparency on PDMS (Figure S3c,d, Supporting Information). The relationship between the linewidth and resistance of the Pt strip on PDMS, with a length of ≈5 mm and a thickness of ≈100 nm, is shown in Figure S6f (Supporting Information). Figure S3e (Supporting Information) illustrates that multiple materials and layers (Steps B1/2 + C1 + D1/2) can be patterned using an aligner machine, see Figure S2D1 (Supporting Information).

Thus, the toolkit demonstrates excellent versatility in fabricating a wide range of materials with superior patterning capability (various materials/substrates and conformal patterning), ultra‐flexibility, high‐density arrays (submicron resolution), high transparency, and assembly of multiple layers/materials. Most importantly, the fabrication processes are cleanroom‐free and solvent‐free (with the exception of water), making them cost‐effective and environmentally friendly. These attributes are crucial for advancing bioelectronics applications in wearables and implants, which will be further discussed with relevant examples in subsequent sections.

## 4 Exemplar Bioelectronics

To showcase the versatility of our toolkit, we validated various wearable and implantable bioelectronics, considering factors such as high‐density arrays, flexibility, surface engineering, and multi‐layer/material geometry, as discussed in the following sections.

### 4.1 High‐Density Array: Implantable Neuroelectrode Arrays (Step B1 + C1 + D3)

High‐density flexible neuroelectrodes play a crucial role in enhancing spatial resolution and minimizing brain tissue damage compared to traditional devices.^[^ ^28^, ^29^ ^]^ In contrast to PaC‐based bioelectronics, PDMS elastomer‐based neuroelectrode implants are more suitable for more invasive applications such as brain penetrating probes because PDMS thickness can be easily adjusted to achieve sufficient stiffness for penetrating through the brain tissue, whereas PaC substrates, being thin and flexible, are challenging to surgically implant without the aid of complex shuttle mechanisms. Meanwhile, PDMS can also be made thin and flexible enough by adjusting the spin coating rate. Through precise control of PDMS substrate thickness, our toolkit enables neuroelectrode arrays to be ultra‐flexible (30 µm thick PDMS substrate) for surface brain recording^[^ ^30^ ^]^ or slightly rigid (130 µm thick PDMS substrate) for deep brain recording.^[^ ^31^ ^]^

**Figure 2:**
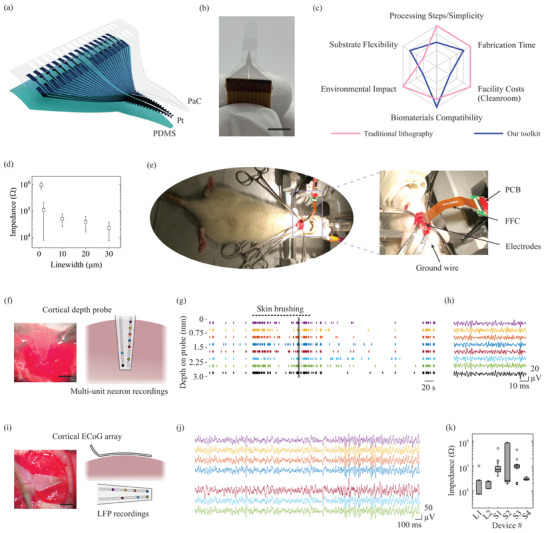
a) Brain neuroelectrode array–device schematic with PDMS substrate, micron‐scale Pt electrodes, and PaC encapsulation layer; b) Real image of the fabricated electrodes integrated with FFC cable (scale bar: 5 mm); c) Comparison of biomaterials/substrate compatibility, fabrication simplicity, and cleanroom requirements between traditional lithography and our fabrication toolkit, specifically for fabricating this neuroelectrode array (see Table S1, Supporting Information for details); d) Electrode linewidth versus impedance (in vitro test, and the error bars represent measurements for three identical samples); e) in vivo experimental setup; f) Image and diagram of device employed as a penetrating probe for cortical recordings (scale bar: 3 mm); g) Raster plot of recordings obtained using device as a cortical penetrating probe, implanted into the sensory cortex of an anaesthetized rat; h) Sample traces recorded from cortical depth probe during skin brushing (grey box in (g), 400–2000 Hz); i) Image and diagram of device employed as an ECoG device on the brain surface (scale bar: 1.5 mm); j) Sample LFP traces recorded from ECoG device implanted on the cortex of an anaesthetized rat (10–300 Hz); k) Box plots of impedance values at 1 kHz for various device implanted into the brain. “L” denotes the large electrode device (300 µm diameter), and “S” denotes the small electrode device (140 µm diameter). n = 7 to 26 functional electrodes per device. Data shown in panels (g,h) and (j) are obtained from “L” (300 µm electrode diameter) devices. Colored electrodes in (f) and (i) represent electrode positions corresponding to the raster plot and traces in (g,h) and (j).

As shown in **Figure** **2**a,b, we patterned 32‐channel arrays with submicron linewidth on PDMS using Step B1 + C1, depositing ≈100 nm thick Pt or Au film via PVD. For Au deposition, a 10 nm Ti seed layer was employed to enhance adhesion to PDMS, in addition to plasma treatment on the PDMS surface. Subsequently, Step D3 was utilized to pattern the PaC encapsulation layer. A 2 µm thick PaC layer was deposited over the metal arrays, and then a WSA mask shaped with a CO_2_ laser cutter was aligned with the metal arrays on the transparent PaC layer to protect specific regions (Figure S2D3, Supporting Information). This was followed by reactive ion etching to expose the electrode areas intended for interaction with brain tissue. After dissolving the WSA mask with water, only the circular electrode area at the end remained exposed while the metal track was encapsulated by PaC. We fabricated a series of neuroelectrode arrays with varying densities, ranging from linewidth of 1–30 µm with a spacing of 40 µm.

Before in vivo experimentation, the fabricated electrode arrays were characterized in phosphate‐buffered saline (PBS) using an Intan RHS stim/record system (see Figure S7, Supporting Information), showing impedance ranging from 23 to 947 kΩ, corresponding to linewidths from 30 to 1 µm (Figure 2d). Notably, electrode arrays with 1‐µm linewidth exhibited impractically high impedance in the in vitro test. Future studies could explore increasing metal array thickness or electrodeposition of conductive polymers such as PEDOT: PSS^[^ ^32^ ^]^ onto the metal arrays to lower impedance. In this study, two types of neuroprobes (100 kΩ impedance with a small electrode pad–140 µm diameter, and 15 kΩ impedance with a large electrode pad–300 µm diameter) were validated through the brain in vivo experiments using a rat terminally anaesthetized model (Figure 2e). The mechanical properties of the designed neuroelectrodes rendered them sufficiently stiff for implantation as penetrating cortical probes (Figure 2f–h) yet flexible enough to allow for good contact as ECoG (electrocorticography) probes (Figure 2i–k). When implanted into or onto the cortex of rats, the devices were capable of recording both multi‐unit neural signals (Figure 2g,h) and local field potential (LFP) activity (Figure 2j). The colored electrodes in diagrams (f) and (i) represent electrode positions corresponding to the raster plot and traces in (g,h) and (j), respectively. For the penetrating probes, physiological stimuli in the form of skin brushing on the contralateral side of the body led to increased firing activity (brushing period indicated by the dashed line in g). The impedances were also tested in vivo for both the large and small electrode devices (large “L” devices 19.53 ± 18.04 kΩ, small “S” devices 118.11 ± 179.32 kΩ, n = 27 and 67 microelectrodes, respectively), with variations potentially arising from the varying track lengths (0.3–3.3 mm) and the contact between the flat flexible cable (FFC) and the metal arrays on PDMS. All these results demonstrate that our toolkit successfully fabricated both depth probe and ECoG neuroelectrode arrays capable of recording a range of neural signals in vivo.

### 4.2 Ultra‐Flexibility: Wrist Pulse Wearables (Step B1 + C1)

Flexibility is crucial for wearable bioelectronics to ensure comfortable skin contact without compromising functionality^[^ ^33^ ^]^ Using our toolkit (Step B1 + C1), we fabricated micron‐scale metal strips via a two‐photon laser source. Uncured PDMS was spin‐coated (1500 rpm) onto a glass slide, and the PaC mask was transferred onto it. After curing, the PaC mask adhered closely to the PDMS, followed by Pt sputtering (≈100 nm thickness) to create a 20 mm × 100 µm strip on PDMS. A carbon‐based fiber wire (ELAT, Fuelcell store) was affixed to the strip end with conductive adhesives using a mixture of PDMS and Ag.^[^ ^34^, ^35^ ^]^ For effective adhesion, the PDMS substrate underwent a 1 min plasma treatment at 100% power and 30% oxygen prior to materials deposition. A thin PDMS layer was subsequently applied atop the strip to enhance flexibility,^[^ ^36^ ^]^ with thickness controlled approximately (≈100 µm thickness) by an air blow gun. The metal strip embedded within the PDMS matrix could then be easily shaped using a blade.

**Figure 3:**
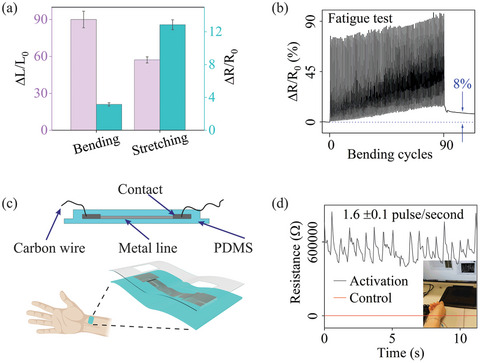
a) Electrical resistance changes with mechanical bending and stretching test; b) Fatigue buckling test under 50% of ∆L/L_0_; c) Wrist pulse wearables–device schematic; d) Wrist pulse test results. The error bars represent measurements for three identical samples. Metal strip: 20 mm length × 100 µm width × 100 nm thickness.

Both mechanical bending and stretching tests were conducted on a strip‐type device (see Figure S8, Supporting Information). The device exhibited mechanical resilience achieving 90% bending and 57% stretching strain (i.e., %strain = displacement/original length, ∆L/L_0_, see **Figure** **3**a). Electrical resistance remained stable upon returning to its original state, indicating excellent flexibility and mechanical stability, due to the effective protection of metal patterns within the PDMS matrix. At ≈67% stretching strain, the PDMS matrix ruptured, yet the metal strip maintained electrical functionality (see Figure S8, Supporting Information). In cyclical fatigue testing (Figure 3b), resistance increased by only 8% after 90 buckling cycles at 50% strain, further validating the flexibility and durability of the metal patterns within PDMS. The strip's electrical resistance changes were recorded when placed on the wrist for pulse measurement; slight stretching with transparent tape secured it to the wrist pulse region (Figure 3c). As a wearable pressure sensor, the change in electrical resistance of the strip indicates mechanical deformation caused by the wrist pulse. Detected pulse signals in Figure 3d indicated a wrist pulse rate, i.e., a heart rate of 96 beats per minute, consistent with normal adult ranges.^[^ ^37^ ^]^

### 4.3 Surface Engineering: Aptamer‐Based Biosensors (Step B2 + C1 + D2)

**Figure 4:**
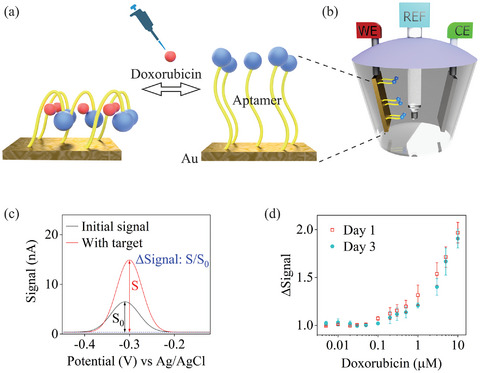
a) Aptamer‐based biosensor mechanism, b) Characterization setup (blue–the methylene blue reporter, yellow–the aptamer base, red–the drug (doxorubicin), WE–working electrode, REF–the reference electrode Ag/AgCl, CE–the counter electrode Pt); c) Electrical signal recording with/without target drug; d) Signal change versus drug concentration. The error bars represent measurements for three identical samples.

Surface engineering is critical in bioelectronics, requiring materials to be chemically stable and resistant. Aptamer‐based biosensors represent a recent advancement in diagnostic technology, employing synthetic nucleic acid molecules to detect target analytes with high precision. Their versatility extends across numerous clinical applications, including early disease detection^[^ ^38^ ^]^ and monitoring therapeutic responses.^[^ ^39^ ^]^ Here, we demonstrate surface engineering using our toolkit by fabricating an aptamer‐based biosensor for doxorubicin drug detection. As depicted in **Figure** **4**a, a methylene blue‐functionalized aptamer was immobilized on an Au surface, interacting with doxorubicin to induce a conformational change that alters electron transfer between the methylene blue reporter and the Au electrode surface, resulting in measurable current changes using square‐wave voltammetry.^[^ ^40^, ^41^ ^]^ Using Step B2 + C1 + D2, we fabricated a device consisting of millimeter‐scale Au patterns on PDMS, featuring a 0.5 mm diameter pad with a 0.1 mm × 8 mm track. Masks were created using a laser cutter, and then transferred onto a PDMS substrate by WSA. Due to poor mask/PDMS contact, dry materials processing like evaporation was used to deposit a thin Au film (≈100 nm) onto PDMS. To enhance adhesion between the Au film and PDMS, the PDMS substrate underwent plasma treatment, and a 10 nm Ti layer was evaporated before Au deposition. The metal track was encapsulated by PDMS or PaC using Steps D2 or D3, allowing only the circular pad to remain exposed.

Before functionalizing the Au pad, we assessed its stability in various solvents. The device exhibited negligible electrical performance change after exposure to acetone, isopropanol alcohol, and ethanol for a week. Subsequently, the device underwent a series of chemical treatments, taking approximately a day, for the chemical functionalization of the Au electrode, using the recipe as described in^[^ ^42^ ^]^ with DNA Oligo (sequence: /5ThioMC6‐D/AC CAT CTG TGT AAG GGG TAA GGG GTG GT/3MeBlN/, *Integrated DNA Technologies, Inc*.). The device was then stored in PBS for additional days. Characterization involved square wave voltammetry at 120 Hz frequency by adding doxorubicin drug in PBS solution with Ag/AgCl and Pt electrodes as reference and counter electrodes, respectively. Figure 4c,d illustrates the device's functionality after chemical processing, showing robust performance with ≈0.1 µM sensitivity to doxorubicin drug. The signal peak ≈−0.3V potential correlated with doxorubicin concentration in the electrolyte. The device maintained consistent performance between the first and third days.

### 4.4 Multi‐Layer/Material Geometry: OECT (Step B1 + C1 + D2)

Our toolkit also demonstrates excellent capabilities in fabricating multi‐layer/material geometry, exemplified by OECT, a fundamental unit in bioelectronics applications such as circuit/neuromorphic/sensing elements.^[^ ^43^ ^]^ The OECT device, featuring a patterned metal layer, semiconductor layer, and encapsulation layer, was fabricated using Step B1 + C1 + D2. Micron‐resolution patterns of the OECT were achieved using a two‐photon laser source. After transferring the PaC mask onto a PDMS substrate, a 300 nm thick layer of PEDOT: PSS was spin‐coated through the PaC mask. Subsequently, the PEDOT: PSS tip was masked (Figure S2D2, Supporting Information) using WSA before metal deposition (≈100 nm thick sputtered Pt or evaporated Au), followed by peeling off the PaC mask. Next, using strategy D2, the PEDOT: PSS tip was masked again with WSA to encapsulate the metal tracks using either PaC deposited via CVD or a photo‐curable elastomer layer via drop‐casting/air‐blown/UV curing. After removing the WSA with water, only the exposed PEDOT: PSS tip (30 µm × 30 µm) remained, while the metal tracks were encapsulated. In contrast to patterning PaC encapsulation using Strategy D3, Method D2 provides faster and simpler processing, though with reduced precision in masking the PEDOT: PSS tip. Precision in the PEDOT: PSS tip was crucially influenced by the WSA masking process, dependent on the aligner and the laser cutter for shaping WSA mask.

**Figure 5:**
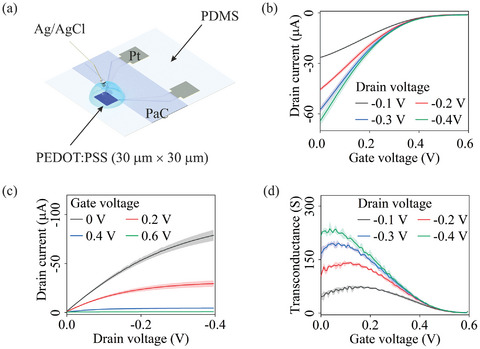
a) OECT characterization setup (the gate electrode is Ag/AgCl; blue represents the PEDOT: PSS; grey indicates the Pt metal track; and there is a PaC encapsulation layer on top of the metal track); b–d) Characterization results of the fabricated OECT. The error bars represent an average from three measurements.

As depicted in **Figure** **5**a, the fabricated V‐shaped OECT was characterized using a standard setup with Ag/AgCl as the gate electrode and 0.1 m PBS as the electrolyte. In Figure 5b–d, output characteristics indicated device operation from 0 (ON) to 0.6 V (OFF) gate voltage, consistent with previous findings.^[^ ^44^ ^]^ These results demonstrate the steady‐state performance of the OECT device fabricated using our toolkit, showcasing successful micron‐resolution OECT fabrication through a cleanroom‐free approach.

## 5 Conclusion

This study proposes a versatile toolkit for fabricating intricate bioelectronics on flexibles with submicron resolution for wearable and implantable applications. The processing techniques include direct laser writing masks, mask transfer, materials deposition, and assembly of multiple materials and layers. These processes offer a versatile toolset for fabricating bioelectronics in a cleanroom‐free environment, enabling the utilization of a diverse range of biomaterials (metal, functional, and encapsulation materials) on flexible substrates such as PDMS, parylene‐C, thin polymer sheets, skin, fabric, and plant leaves. Our toolkit demonstrates a strong potential to replace traditional lithography for the fabrication of implantable‐scale bioelectronics with micron resolution. As shown in Figure 2 c (see Table S1, Supporting Information), our toolkit offers advantages in terms of time, procedure, cost, environmental impact, and compatibility with a wider range of biomaterials, as compared to traditional photolithography. Ongoing research aims to optimize the laser scanning speed and power to minimize the duration of the laser writing mask. Our toolkit demonstrates excellent capabilities with superior patterning precision (1 µm linewidth), ultra‐flexibility (90% bending and 57% stretching strain), high density (29 µm spacing), and the ability to create complex geometries. Conformal deposition is also demonstrated, allowing for patterning across both edge and curved surfaces. The toolkit's effectiveness was validated through several bioelectronics examples, showcasing the material merits enabled by this fabrication toolkit. The ultra‐flexibility merit was successfully applied to a wearable pulse wristband, which recorded a heart rate of 96 beats per minute. Surface engineering was demonstrated by fabricating an aptamer‐based biosensor for doxorubicin drug detection, with the device showing robust performance and ≈0.1 µM sensitivity to the doxorubicin drug. The ability to fabricate intricate geometries was exemplified by a V‐shaped OECT device with a 30 µm × 30 µm channel, demonstrating steady‐state performance. Additionally, high‐density neuroelectrode arrays were fabricated and successfully applied to brain recordings in vivo, for both penetrating and ECoG probes.

In conclusion, our toolkit demonstrates excellent versatility for fabricating submicron‐resolution bioelectronics with intricate geometries for both wearable and implantable applications. This straightforward cleanroom‐free toolkit has the potential to replace lithography and become a dominant fabrication technique in the field of bioelectronics.

## 6 Experimental Section

### Fabrication

1. Step A: A two‐photon laser (Nanoscribe) or a CO_2_ laser (VLS, Universal) was utilized for direct writing of the mask materials, e.g., PaC and polyimide sheet. Approximately 2 µm thick PaC film was deposited on a soaped glass substrate (1:5 mixture of Decon 90 and DI water, dried by air blowing) via CVD (PDS 2010 Labcoter). The soap treatment facilitated the peel‐off process. For the CO_2_ laser, parameters were set to 6% speed, 6% power, and 1000 PPI. The total applied power of the two‐photon laser was controlled by adjusting the number of slices, determining the extent of laser ablation. In this case, the two‐photon laser parameters were fixed at 17 slices, 0.1 hatching, 100 k scan speed, and 100% power. The pattern was designed using AutoCAD.
2. Step B: WSA (PVA‐based 3M 5414 tape) was employed for mask transfer. PDMS (1: 10 of cure agent and the base) of varying thicknesses was spin‐coated on a glass slide at speeds ranging from 500 to 1500 rpm. The PaC mask was placed onto the uncured PDMS and then degassed for ≈8 min using an Edwards rotary pump to eliminate air gaps between the PaC and the PDMS. Subsequently, the PDMS was cured at 150 °C for 25 min. After removing the soaped glass substrate, the PaC mask remained adhered to the PDMS.
3. Step C: Regarding materials deposition, a PEDOT: PSS solution was prepared and spin‐coated (Laurell Technologies) on PaC/PDMS using a commercially available product (Clevios PH1000), which was mixed with 5 vol% ethylene glycol, 0.25 vol% DBSA, and 1 vol% GOPS as a crosslinker, following the previously established recipe.^[^ ^45^ ^]^ For dry processing, ≈100 nm thick films of Au with a 10 nm thick Ti seeding layer were deposited using an electron beam evaporator (Kurt J. Lesker), while ≈100 nm thick Pt films were deposited using a sputtering kit (Emitech K575X, Quorum Technologies Ltd.). The conductive eutectogel^[^ ^46^ ^]^ remained solid state at room temperature and melted at 80 °C, a temperature at which the WSA retained its functionality, making the gel suitable for blade printing processing.
4. Step D: WSA facilitated the mask transfer process and served as the mask for subsequent additive or subtractive processes. Experimental results confirmed that WSA remains water‐soluble after PVD (sputtering and evaporation) and reactive ion etching (PlasmaPro 80, Oxford Instruments, using 50 sccm O_2_, 8 sccm CF_4_, 2 sccm SF_6_, and 180 W plasma power). The etching rate of reactive ion etching was measured at ≈170 nm min^−1^ for PaC. Alignment was achieved using a micron‐resolution aligner (Picoplacer, Finetech GmbH & Co. KG).

The final step involves integrating the fabricated device with cables. For devices with a large contact area and spacing, a mixture of PDMS and Ag nanoparticles (Asahi Kagaku, PN: LS453‐6B) was utilized to manually join a carbon‐based fiber wire (ELAT, Fuelcell store) with the contact region, followed by PDMS encapsulation. Small devices, such as neuroelectrode arrays, were connected using a flat flexible cable – FFC (15015‐0233, Molex) and tape (3T‐TGP20500N, 3T Frontiers Pte Ltd), aligned using the Finetech bonder machine.

### Characterisation

The electrical resistance of the metal strip was characterized using a probe station equipped with a Keysight meter/source (two‐probe method). The sample thickness was measured using a stylus profilometer (Dektak, Bruker). SEM imaging was conducted using a Leo 1530 VP system (Gemini), while optical microscopy utilized an Elipse LV 100ND (Nikon Metrology NV). Mechanical testing involved a linear motor (LinMot, PS01‐23 × 80‐R stator) to control device buckling/stretching, with resistance continuously monitored via a Keithley 2110 digital multimeter (two‐probe method). The cyclical fatigue test was carried out at 0.05 Hz with a motion range of 5 mm (maximum speed: 0.5 mm s^−1^; acceleration and deceleration: 10 m s^−2^). Square wave voltammetry was employed to characterize the aptamer‐based device using a PalmSens 4 potentiostat in a three‐electrode setup, with a Pt counter electrode and an Ag/AgCl reference electrode in a coiled wire (IJ Cambria Scientific Ltd.). The OECT device was characterized using a semiconductor device analyzer (Keysight B1500A), with an Ag/AgCl pellet (World Precision Instruments) serving as the gate electrode inside a Faraday cage under ambient conditions (40% relative humidity at 20 °C) and ambient light. The fabricated neuroelectrode arrays were evaluated in vitro in PBS solution by connecting the implant to a 32‐channel stim/recording head stage (Intan Technologies) via a custom printed circuit board (PCB).

Wearable experiments were performed under the approval of the Ethics Committee of the Department of Engineering at the University of Cambridge (6/9/2018, IONBIKE).

In vivo validation was carried out in accordance with the UK Animals (Scientific Procedures) Act, 1986. Animal work was approved by the Animal Welfare and Ethical Review Body of the University of Cambridge and by the UK Home Office (project license number PFF2068BC). Surgical implantation was carried out on Sprague Dawley rats ≈150g in weight (Charles River, UK) under terminal urethane anesthesia (1.2 g kg^−1^ i.p.), with body temperature monitored and maintained using a thermal blanket. Rats were group‐housed in individually ventilated cages with ad libitum access to food and water prior to implantation. Animals were mounted onto a stereotaxic frame, their skull exposed, a cranial window drilled above the sensory cortex, and the dura within the window removed. When operated as a cortical depth probe, a neuroelectrode array was mounted onto the stereotaxic frame and directly lowered into the exposed cortex to a maximum depth of 3 mm. When operated as a cortical ECoG array, a neuroelectrode array was simply placed on top of the exposed cortex. The device was then connected to an electrophysiology acquisition system (RHS stim/recording system, Intan Technologies) through a custom PCB. During cortical depth probe recordings, the skin of the contralateral side of the animal was brushed by the experimenter to trigger cortical sensory activity. A steel wire and screw previously implanted above the cerebellum of the animal was also connected and used as ground. Data was then amplified and sampled at a rate of 30 kHz. Impedance values (at 1 kHz) for functioning electrodes were also acquired during implantation. Data analysis and plotting were performed in Matlab (R2023a, Mathworks). The analysis involved down sampling of the acquired traces and filtering between either 400–2000 Hz (cortical probe, 6th order Butterworth bandpass filter) or 10–300 Hz (ECoG array, 2nd order Butterworth bandpass filter), in addition to a notch filter to eliminate mains noise. For cortical probe recordings, spikes were identified as negative peaks using a threshold of 5 times the trace standard deviation (≈12 µV).

The repeatability of this toolkit could be evaluated using neuroelectrode arrays, as shown in Figure 2d, where each data point represents impedance measurements from three identical samples. A total of 15 devices are presented in Figure 2d, and six devices are shown in Figure 2k, comprising 21 devices randomly selected from two fabrication batches. The error bars in Figure 2d represented device‐to‐device impedance variations, capturing all fabrication‐related inconsistencies in the toolkit, such as alignment precision for the PaC encapsulation layer, interface resistance introduced by FFC cable bonding, and material performance differences between fabrication batches. Meanwhile, the error bars in Figure 2k reflected impedance variations across electrodes on the same device, resulting from factors such as alignment accuracy for the PaC encapsulation layer and differences in track lengths of individual electrodes on the device.

## Conflict of Interest

The authors declare no conflict of interest.

## Supporting information



Supporting Information

## Data Availability

The data that support the findings of this study are available from the corresponding author upon reasonable request.
