# Supplementary material for: Cleanroom‐Free Toolkit for Patterning Submicron‐Resolution Bioelectronics on Flexibles
Source: Small. 2025 Mar 7;21(14):2411979. doi: 10.1002/smll.202411979 (PMC11983252; doi:10.1002/smll.202411979)
Supplement: Supplementary file 1 — Supporting Information [file SMLL-21-2411979-s001.pdf]

NANO · MICRO

small

Supporting Information

for *Small*, DOI 10.1002/smll.202411979

Cleanroom-Free Toolkit for Patterning Submicron-Resolution Bioelectronics on Flexibles

*Xudong Tao, Alejandro Carnicer-Lombarte, Antonio Dominguez-Alfaro, Luke Gatecliff, Ji Zhang, Sophia Bidinger, Scott T. Keene, Salim El Hadwe, Chaoqun Dong, Alexander J. Boys, Christopher Slaughter, Ruben Ruiz-Mateos Serrano, Jakob Chovas, Marco Vinicio Alban-Paccha, Damiano Barone, Sohini Kar-Narayan and George G. Malliaras**

Supporting Information

Cleanroom-Free Toolkit for Patterning Submicron-Resolution Bioelectronics on Flexibles

*Xudong Tao[1], Alejandro Carnicer-Lombarte[1], Antonio Dominguez-Alfaro[1], Luke Gatecliff[1], Ji Zhang[2], Sophia Bidinger[1 3], Scott T. Keene[4], Salim El Hadwe[1 8], Chaoqun Dong[1], Alexander J. Boys[5 6], Christopher Slaughter[1], Ruben Ruiz-Mateos Serrano[1], Jakob Chovas[3], Marco Vinicio Alban-Paccha[1 7], Damiano Barone[1 8 9], Sohini Kar-Narayan[2], George G. Malliaras[1]**

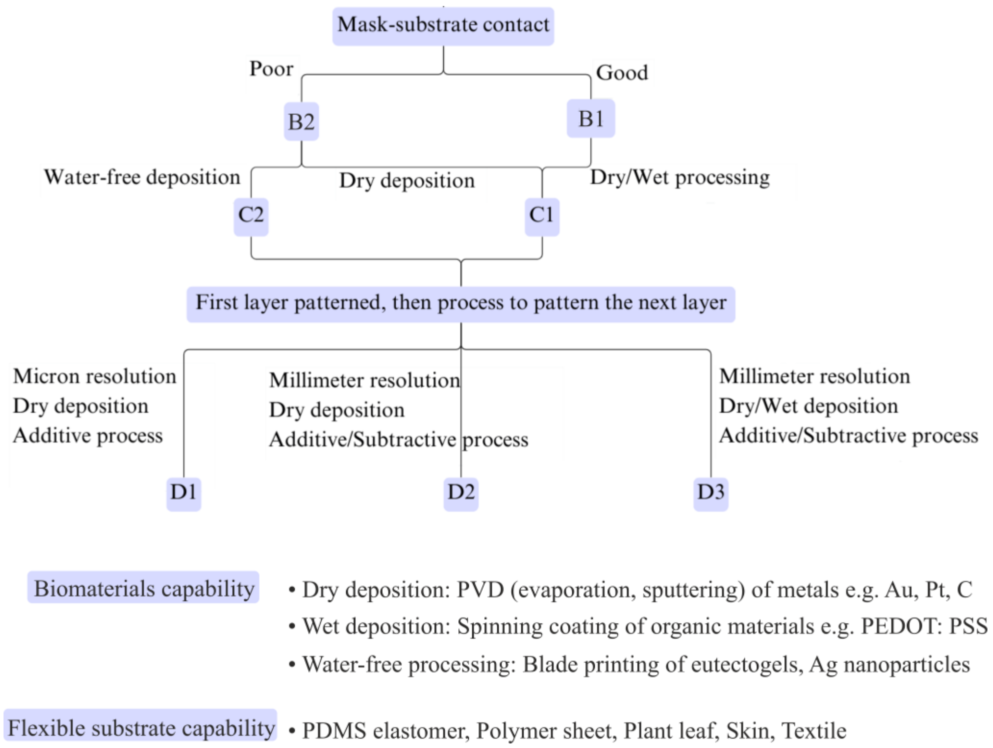

Figure S1. Flowchart for step-by-step selection with the biomaterials and flexible substrates demonstrated in this study.

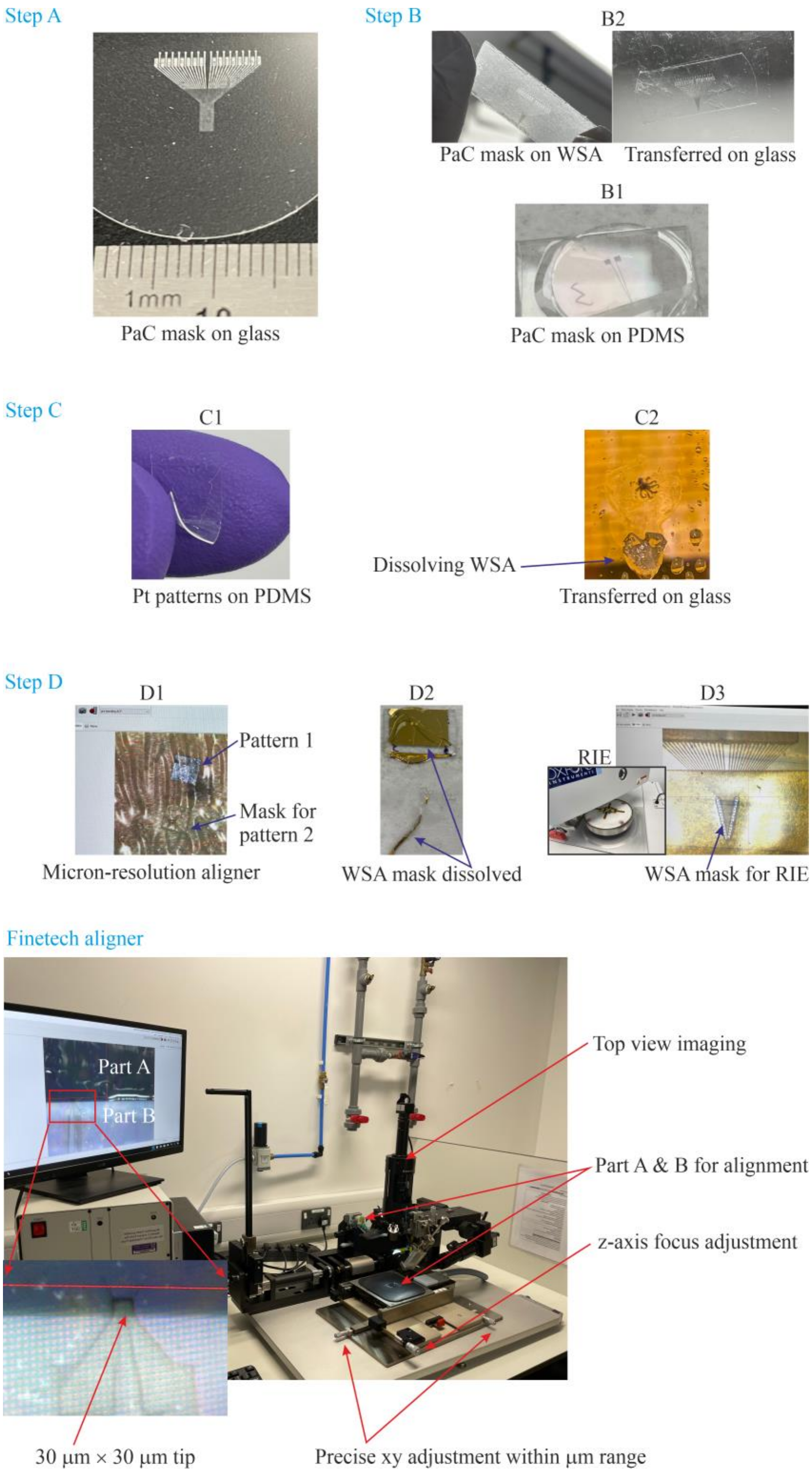

Figure S2. Toolkits corresponding to Figure 1, along with an image of the micron-resolution aligner.

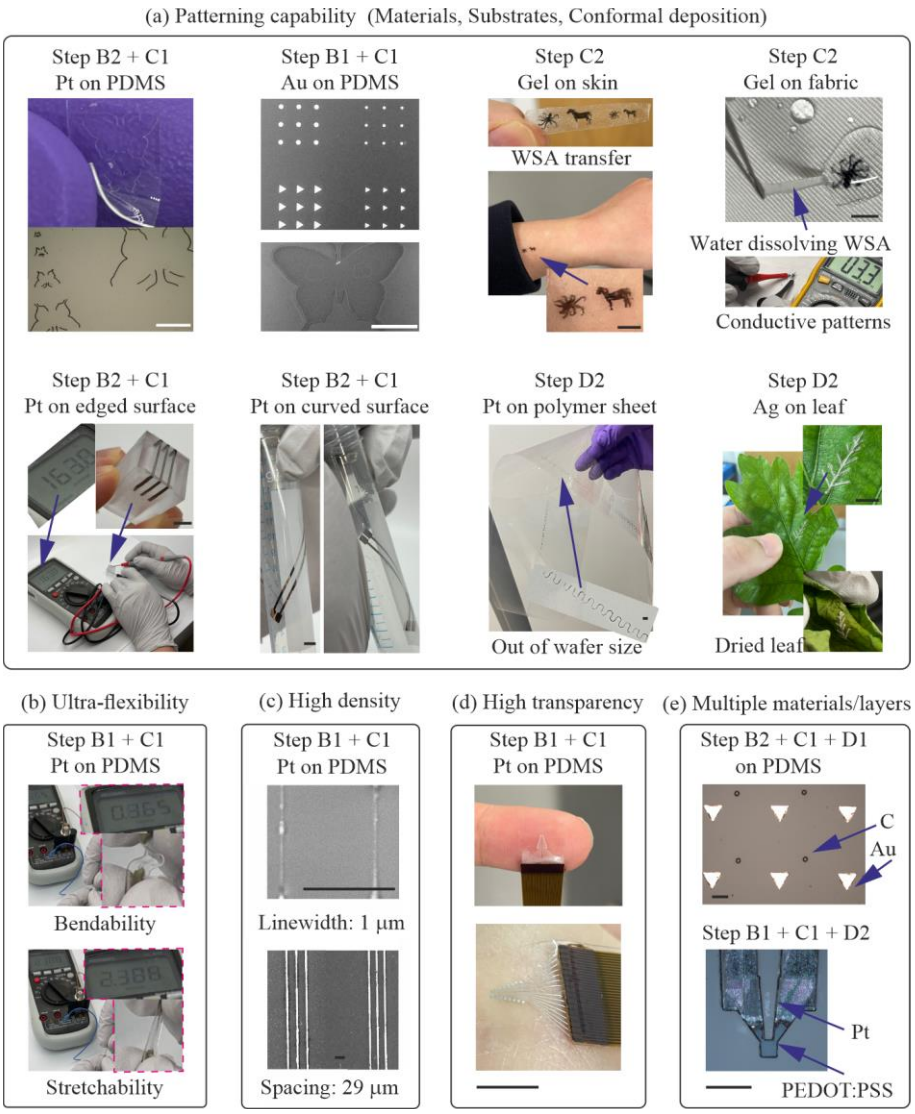

Figure S3. Materials properties: (a) Patterning capability of different materials on different substrates (white scale bar: 500 µm. black scale bar: 5 mm); (b) High flexibility with electrical functionality (metal linewidth: 100 µm); (c) High density with submicron resolution (scale bar: 50 µm); (d) High transparency arrays (scale bar: 6 mm); (e) Multiple layers and materials (scale bar: 100 µm).

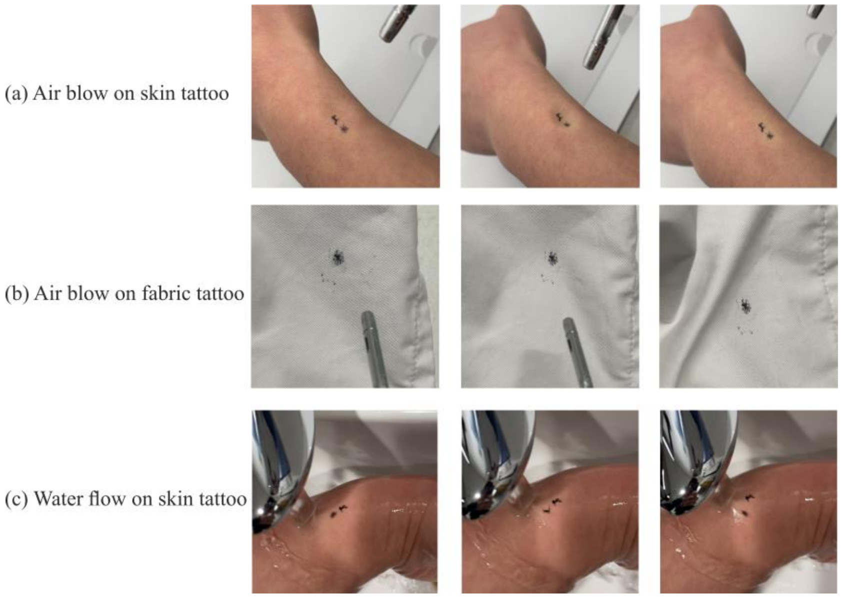

Figure S4. (a & b) Air blow resistance of the gel on skin and fabric; (c) Water flow resistance of the gel on skin.

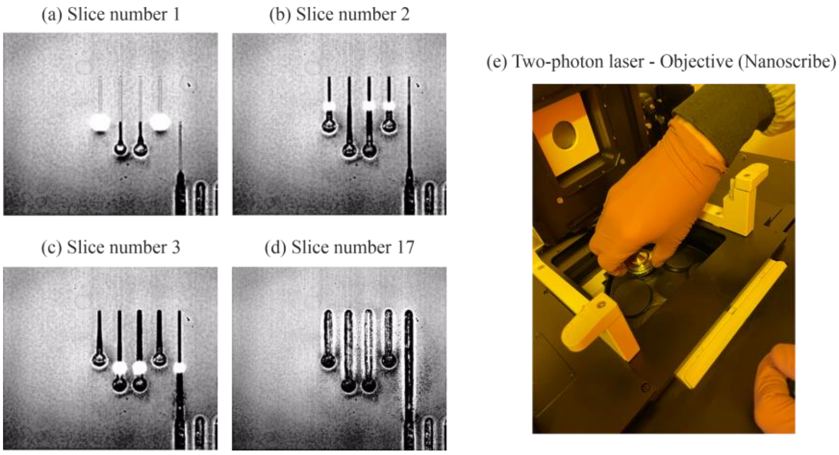

Figure S5. (a-d) Two-photon laser ablation on PaC with different slice numbers; (e) Objective in Nanoscribe.

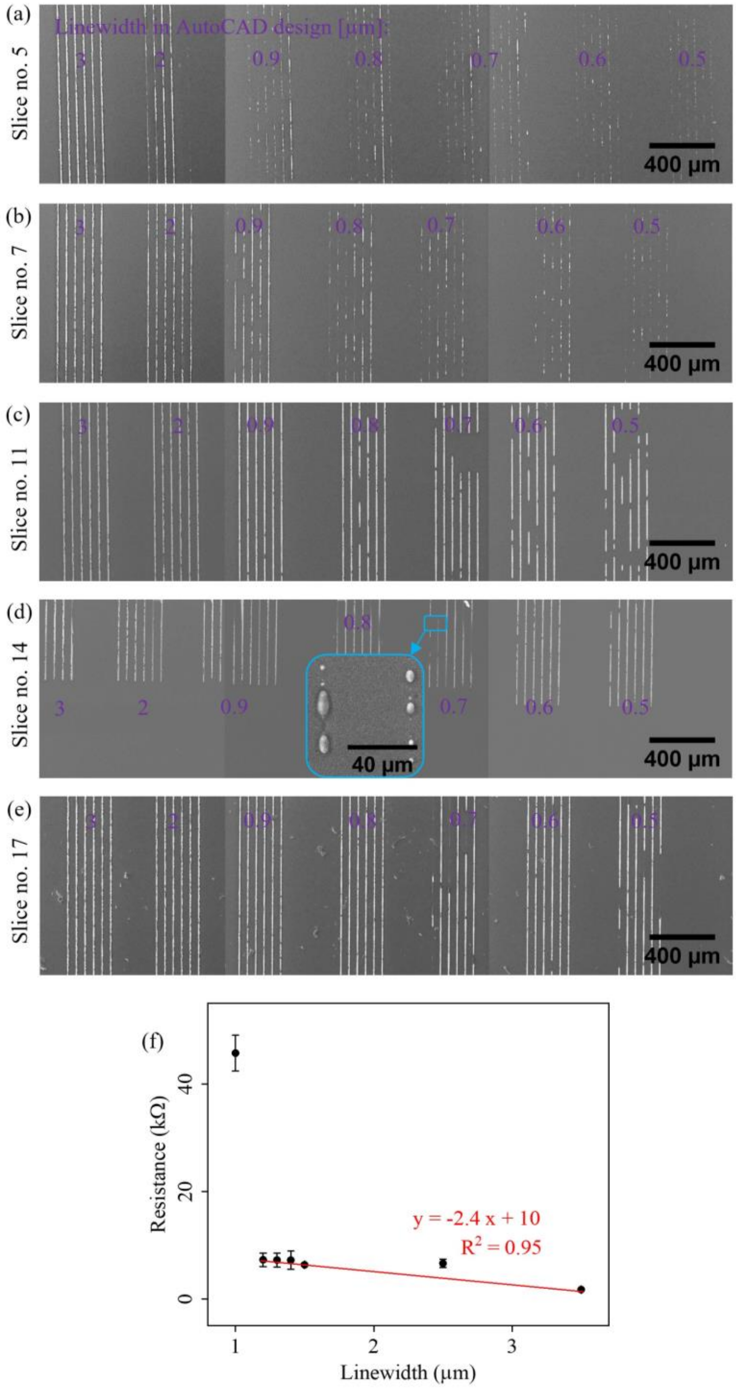

Figure S6. (a-e) SEM images of Pt arrays fabricated by different slice numbers and linewidth design; (f) Relation between the linewidth and electrical resistance.

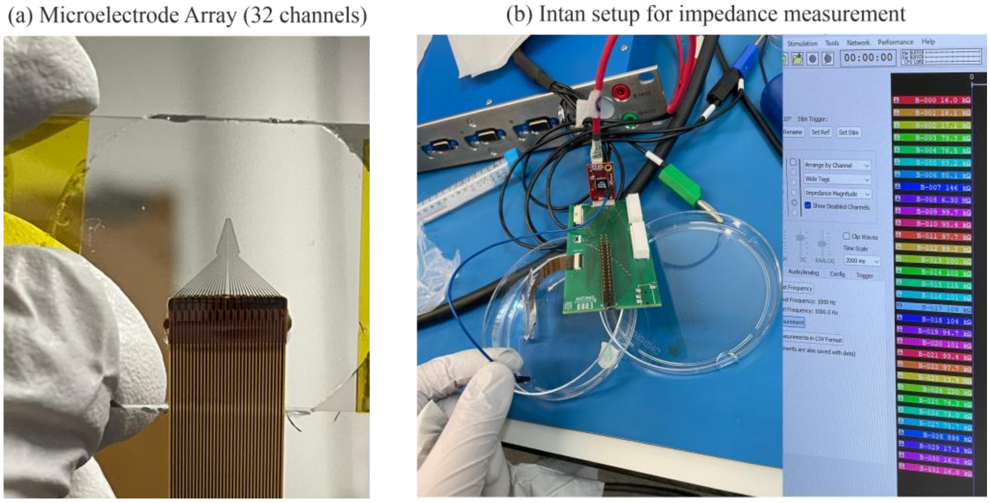

Figure S7. (a) Image of neuroelectrode arrays with 10-μm linewidth; (b) Intan recording of resistance of neuroelectrode arrays with 2-μm linewidth at the tip.

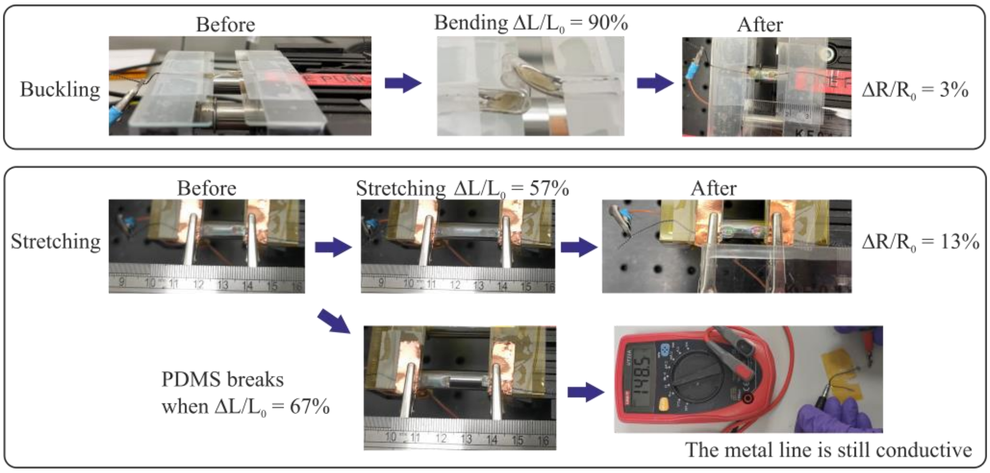

Figure S8. Mechanical characterization of the metal strip in PDMS matrix.

Table S1. A comparison between photolithography and our toolkit, in the case of fabrication of neuroelectrode arrays, as illustrated in Figure 2 a. The estimated time for lithography is derived from researchers' perspectives at universities or institutions, rather than from an industrial viewpoint where these processes are typically automated by machines.

	Photolithography	Estimated time		Our toolkit	Estimated time
	Mask design	-		Mask design	-
	Mask order	1 week			
**1st layer for metal pattern**	(1) PaC deposition on Si wafer	-		(1) PaC deposition on glass	-
	(2) Substrate preparation	3 min		(2) Laser writing PaC mask	50 min
	(3) Photoresist application (spin coating/soft bake)	20 min			
	(4) Exposure	10 min			
	(5) Post-bake	2 min		(3) Mask transfer (Solution B1)	20 min
	(6) Development	5 min			
	(7) Hard bake	5 min			
	(8) Materials deposition	-		(4) Materials deposition	-
	(9) Lift-off	30 min		(5) Peel off mask	1 min
**2nd layer for encapsulation pattern**	PaC deposition	-		PaC deposition	-
	Photoresist application (spin coating/soft bake)	10 min		Laser cut WSA (Solution D3)	2 min
	Exposure	10 min		Alignment	5 min
	Development	5 min			
	Reactive ion etching	-		Reactive ion etching	-
	Lift-off	10 min		WSA removal	2 min
**The total time**		110 min			80 min
**Characteristics**	➢ Mask order is costly and requires lead time;<br>➢ Many processes are engaged, and each one demands manual handling;<br>➢ Photoresist/solvents/developers could raise environmental issues and may not be compatible with certain biomaterials;<br>➢ Cleanroom conditions are required;<br>➢ PaC substrate with a thickness range of 1 to 5 µm.			➢ Laser writing duration needs to be further improved;<br>➢ Fewer steps are involved, resulting in less manual processing;<br>➢ It avoids utilizing photoresist/solvents, making it compatible with certain sensitive biomaterials;<br>➢ Processing is entirely dry at each step, using only water for WSA removal;<br>➢ PDMS substrate with a thickness range of 5 µm to mm.	
